# LncRNA HOXD-AS1 promotes the metastasis of human hepatocellular carcinoma via modulating miR-326/SLC27A4

**DOI:** 10.1186/s12935-020-01217-8

**Published:** 2020-05-12

**Authors:** Wenbin Ji, Qunfeng Wang, Jian Yang

**Affiliations:** 1 Department of Radiology, Affiliated Taizhou Hospital of Wenzhou Medical University, Taizhou, 317000 Zhejiang China; 2 Institute of Integrated Traditional Chinese and Western Medicine, Gansu University of Traditional Chinese Medicine, Lanzhou, 730000 China; 3 Imaging department, the First Affiliated Hospital of Xi’an Jiaotong University, Xi’an 710061, China

**Keywords:** HCC, HOXD-AS1, Metastasis, miR-326, SLC27A4

## Abstract

**Background:**

Mounting evidences have indicated that long non-coding RNA (lncRNA) HOXD cluster antisense RNA 1 (HOXD-AS1) is dysregulated and participates into the progression of cancers. This study aims to investigate the biological roles and mechanisms of HOXD-AS1 in the metastasis of hepatocellular carcinoma (HCC).

**Methods:**

The quantitative real-time PCR (qPCR) assay was used to assess the level of miR-326 and HOXD-AS1 in HCC tissues and cell lines. The growth of HCC cell was analyzed by using CCK-8 assay and colony formation assay. The migration and invasion of HCC cell were investigated by using wound healing and transwell invasion analysis. The expressions of SLC27A4, N-cadherin and E-cadherin were determined by western blotting. The growth of HCC cell in vivo was assessed by using xenograft model.

**Results:**

Here, we elaborated that HOXD-AS1 was overexpressed in HCC tissues than that in the adjacent normal tissues and the level of HOXD-AS1 was related with the aggressive phenotypes of HCC. Functionally, downregulation of HOXD-AS1 repressed the proliferation, invasion abilities of HCC cell in vitro and the distant metastasis of HCC cell in vivo. Further investigations demonstrated that HOXD-AS1 directly bound with miR-326 and thereby regulated its endogenous target gene, solute carrier family 27 member 4 (SLC27A4).

**Conclusions:**

All these findings indicated that HOXD-AS1-miR-326-SLC27A4 axis participated into the progression of HCC.

## Background

Hepatocellular carcinoma (HCC) is one of the most common and the leading cause of cancer-related deaths worldwide [1]. Although significant advances in the treatment of HCC have been made, the prognoses of patients with HCC are still unsatisfactory. Cancer cell diffusion and metastasis remain the mainly causes of death in patients with HCC, and the process of metastasis is sophisticated which involves a sequence of complex epigenetic and genetic variations. Hence, it is urgently needed to explore the underlying mechanism which drives the metastasis of HCC.

Meanwhile, increasing reports have indicated that lncRNAs are involved into cancer progression and can be considered as prognostic indicators among different cancers, including pancreatic cancer, gastric carcinoma (GC) and non-small cell lung cancer (NSCLC). For instance, H19 reduces the cell viability, mobility, and invasion abilities of thyroid cancer cell through downregulating insulin receptor substrate 1 (IRS-1) [2]. In colorectal cancer, RP4 completely bind with miR-7-5p and regulates the apoptosis and growth of colon cancer cell [3]. Recently, HOXD-AS1 has been identified as an oncogene and enhances the epithelial-mesenchymal transition (EMT) process of breast carcinoma cell through serving as a competing endogenous RNA (ceRNA) for miR-421 [4]. In ovarian carcinoma, HOXD-AS1 promotes the proliferation and invasion of cancer cell through modulating miR-133a-3p level and Wnt signaling axis [5]. In human gastric cancer (GC), HOXD-AS1 silencing inhibits cancer cell growth through inactivating janus kinase 2/signal transducer and activator of transcription 3 (JAK2/STAT3) [6]. All these investigations have proven that HOXD-AS1 serves as an oncogene in multiple tumor types.

MicroRNAs (miRNAs), which are small noncoding RNA, post-transcriptionally modulate the expressions of their target genes. Accumulating studies have firmly established miRNAs as the critical players which regulate many aspects of cancer progression, including growth and metastasis [7]. For example, miR-326 influences the aggressive ability of lung cancer cell through regulating paired-like homeobox 2a (phox2a) [8]. Previous investigations also suggest that the dysregulation of miR-326 contributes to multiple tumor types, such as cholangiocarcinoma, pancreatic ductal adenocarcinoma and medulloblastoma [8–10]. In osteosarcoma, small nucleolar RNA host gene 1 (SNHG1) regulates the level of NIN1 binding protein 1 homolog (NOB1) by sponging miR-326 and promotes the tumorigenesis of osteosarcoma cell [11]. In addition, downregulation of HOX transcript antisense RNA (HOTAIR) inhibits the malignant phenotypes of glioma cell through regulating miR-326 [12]. However, the role of HOXD-AS1-miR-326 axis in the metastasis of HCC hasn’t been reported.

Herein, we proved that HOXD-AS1 was markedly overexpressed in HCC tissues and HCC cells. Upregulation of HOXD-AS1 accelerated the growth, invasiveness, tumorigenesis in vitro, and distant metastasis abilities of HCC cells in vivo. Further investigations suggested that HOXD-AS1 exerted its impacts through binding with miR-326 and future modulating the level of solute carrier family 27 member 4 (SLC27A4).

## Materials and methods

### HCC tissues

A total of 48 cases of HCC and normal samples were collected from patients who received operative treatment for HCC at the First Affiliated Hospital of Xi’an Jiaotong University. None of patients had been treated before operative treatment. The written informed consent was obtained from patients. Our study was approved by the Research Ethics Committee (REC) of the First Affiliated Hospital of Xi’an Jiaotong University. All tissues were snap-frozen in liquid nitrogen and were stored at -80 °C.

### Cell lines

Human HCC cell lines (BEL7402, HuH7, HepG2, SMMC-7721) and normal hepatocytes cell line LO2 were obtained from Nanjing Cobioer Biotechnology Co., Ltd (Nanjing, Jiangsu, China). HEK-293T cell was obtained from Nanjing Cobioer Biotechnology Co., Ltd (Nanjing, Jiangsu, China). HCC cells were cultured in 1640 + 10% FBS, streptomycin (100 μg/ml) and penicillin (100 μg/ml). LO2 and HEK-293T cells were cultured in DMEM (Thermo Fisher Scientific, Waltham, MA, USA).

### Cell transfections

MiR-326 or miRNA negative control (miR-NC), miR-326 inhibitor and its negative control (miR-NC inhibitor) were purchased from Genecopoeia (Rockville, MD, USA). SiRNA (si-HOXD-AS1), SLC27A4 siRNA (siSLC27A4) and negative control (siCon) were purchased from GenePhama (Shanghai, China). Cells were transfected with siRNA by using Lipofectamine™ 3000 (Thermo Fisher Scientific, Waltham, MA, USA). Human HOXD-AS1 transcript cDNA was constructed into pcDNA3.1 vector (GenePhama, Shanghai, China). Lentiviral small hairpin RNA (shRNA) targeting HOXD-AS1 (sh-HOXD-AS1) or negative control (sh-NC) was cloned into pLVshRNA-Puro vector (GenePhama, Shanghai, China). The viruses were packaged in HEK-293T cell and the virus particles were harvested 72 hours later. HepG2 cell was transfected with virus particles and selected using puromycin.

### Cell proliferation assay

Cells were plated into 96-well plates and were maintained at 37 °C. Cell Counting Kit-8 (CCK-8) solution was added into well plates after 24 hours, 48 hours or 72 hours, respectively. The spectrophotometric absorbance was measured at 450 nm.

### Clone formation analysis

1 × 10^3^ cells were cultured into a six well plate and maintained for 14 days. After two weeks, colonies in plate were fixed with paraformaldehyde and stained by using crystal violet (1%) for 15 min. The number of cell colonies (more than 50 cells) was counted.

### Cell migration

Firstly, cells were plated into 6 well plates for overnight. After that, an artificial wound was made by using a 100 μl pipette tip. After washing with PBS, cells were cultured with FBS free medium for 24 hours. The pictures of wound width were taken at 0 hour or 24 hours [13].

### Cell invasion

The 24-well transwell chamber (8 μm; Corning, NY, USA) was precoated with 1:4 diluted Matrigel. 200 μl of cell suspension (1 × 10^4^) was plated into the upper chamber. In the lower chamber of transwell, 600 μl of culture medium + 20% FBS was added. After 18 hours, the invaded cell was stained with crystal violet (1%). The number of invaded cell was counted from five random fields [14].

### Luciferase reporter gene assay

HEK-293T cell was cotransfected with miR-326 and luciferase reporter vector that containing wild type (wt) SLC27A4 3′-UTR or mutant type (mut) SLC27A4 3′-UTR by using Lipofectamine™ 3000. To explore HOXD-AS1′s influence on miR-326, HEK-293T cell was cotransfected with miR-326 and luciferase reporter vector that containing wt HOXD-AS1 sequence or mut HOXD-AS1 sequence. After 48 hours, the luciferase activity in HEK-293T cell was analyzed using Luciferase reporter assay system (Promega, Madison, WI, USA).

### Quantitative real-time PCR (qPCR)

RNAs were isolated from clinical tissues or cultured cells by using Trizol kit (Thermo Fisher Scientific). The cDNA was constructed from 1 μg of RNA by using PrimeScript RT-polymerase (Thermo Fisher Scientific). The qPCR reactions of target genes were conducted using SYBR Green Master Mix (Applied Biosystems, Foster City, CA, USA). MiRNAs were isolated from cells or tissues using a Qiagen miRNeasy FFPE Kit (Qiagen, Hilden, Germany). The cDNA was synthesized using a TaqMan miRNA Reverse Transcription Kit (Applied Biosystems, Foster City, CA, USA). The level of miR-326 was detected using the TaqMan miRNA Assay (Applied Biosystems, Foster City, CA, USA) on 7500 Real-Time PCR System (Applied Biosystems, Foster City, CA, USA). The comparative cycle threshold (CT) method was chosen to detect the levels of target genes or miRNAs through calculating the 2^(-∆∆Ct)^ method. The primer sequences in this study were as follows: SLC27A4: sense: 5′-GGACCCAGGTGGGATTCTC-3′, antisense: 5′-CGCGCCTGATGGTCTTGAT-3′; GAPDH: sense: 5′-GGAGCGAGATCCCTCCAAAAT-3′, antisense: 5′-GGCTGTTGTCATACTTCTCATGG-3′; E-cadherin: sense: 5′-ATTTTTCCCTCGACACCCGAT-3′, antisense: 5′-TCCCAGGCGTAGACCAAGA-3′; N-cadherin: sense: 5′-TTTGATGGAGGTCTCCTAACACC-3′, antisense: 5′-ACGTTTAACACGTTGGAAATGTG-3′. GAPDH or U6 snRNA was used as an endogenous control.

### Immunoblotting

Total proteins were lysed using ice-cold RIPA buffer. Equal amounts of cell lysates (30 μg) were loaded on 10% SDS-PAGE and transferred onto PVDF membranes. After membranes were blocked with 5% skim milk, the PVDF membranes were incubated with SLC27A4 (Beyotime Biotechnology, Nanjing, Jiangsu, China), E-cadherin (Beyotime Biotechnology), N-cadherin (Beyotime Biotechnology) or GAPDH antibody (Beyotime Biotechnology) at 4°C for overnight. After that, PVDF membrane was incubated with HRP-conjugated secondary antibody for 2 hours (Beyotime Biotechnology). The bands were detected by using a chemiluminiscence (ECL) detection system.

### Tumorigenesis and metastasis assay

100 μl of HepG2 cells (2 × 10^6^) were subcutaneously inoculated into BABL/c athymic nude mice (4-5 week-old, Slake Experimental Animal Co., Ltd, Shanghai, China) (n=6 for each group). The tumor volumes were recorded each week. Mice were sacrificed after 35 days, and tumor tissues were collected and weighted. In experimental lung metastasis analysis, 100 μl of HepG2 cells (1 × 10^6^) were injected into BABL/c mice through tail vein (n=6 in each group). After 30 days, all mice were sacrificed and the lungs were collected and applied for hematoxylin-eosin (H&E) staining assay. These animal experiments were approved by the Institutional Animal Care and Use Committee (IACUC) from the first affiliated hospital of Xi’an Jiaotong university.

### Statistical analysis

Data were presented as Mean ± SD. All the experiments were analyzed by using GraphPad Prism 7.0. Kaplan-Meier method was carried out for survival analysis. The student’s t-test was applied to analysis the difference between two groups. *P*<0.05 was considered statistically significant.

## Results

### HOXD-AS1 is overexpressed in HCC

**Fig. 1:**
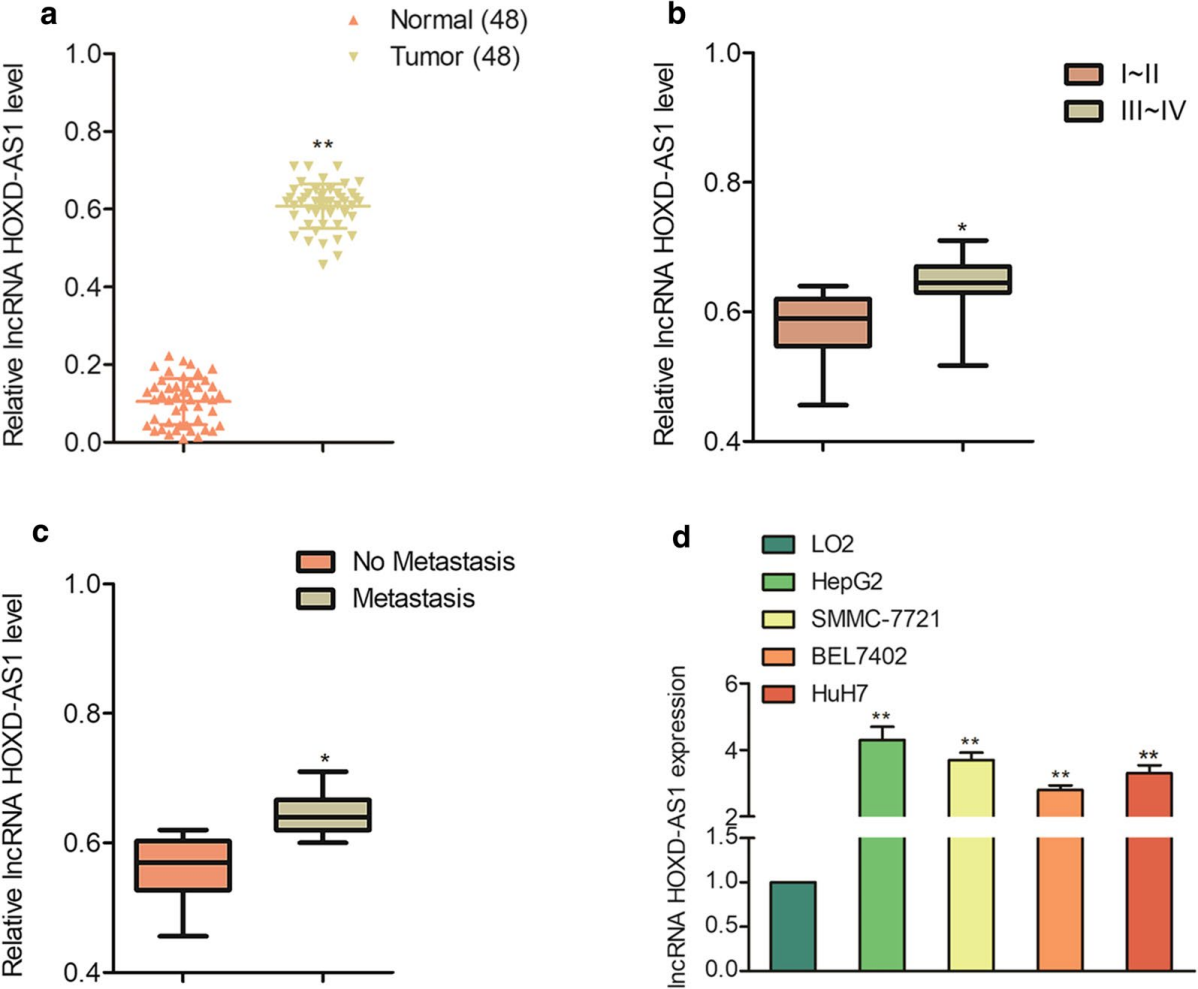
HOXD-AS1 is upregulated in HCC.** a** The levels of HOXD-AS1 in HCC and normal tissues were measured using qPCR. ^**^P<0.01 compared to normal. ** b** The level of HOXD-AS1 was significantly higher in III~IV stage than that in I~II stage. ^*^P<0.05 compared to I-II. ** c** The levels of HOXD-AS1 in HCC tissues without and with distant metastasis. ^*^P<0.05 compared to no metastasis. ** d** The levels of HOXD-AS1 in HCC cells (HepG2, SMMC-7721, BEL7402 and HuH7) and normal hepatocytes cell line, LO2 were detected using qPCR assay. ^**^P<0.01 compared to LO2 cell

Firstly, we detected the levels of HOXD-AS1 in normal tissues (n=48) and HCC tissues (n=48) by using qPCR analysis. As showed in Fig. 1a, HOXD-AS1 was markedly overexpressed in HCC tissues compared to that in the normal tissues. Future analysis indicated that HOXD-AS1 was markedly overexpressed in III~IV stage than that in I~II stage (Fig. 1b). Additional, HOXD-AS1 was markedly overexpressed in HCC tissues with metastasis in contrast to tissues without metastasis (Fig. 1c). Consistently, HOXD-AS1 was upregulated in HCC cells (SMMC-7721, HepG2, HuH7 and BEL7402) than that in normal hepatocytes cell line, LO2 (Fig. 1d). Furthermore, patients with higher HOXD-AS1 level exhibited shorter overall survival than those with lower HOXD-AS1 level ( Additional file 1: Figure S1). Finally, we investigated the relationship between the level of HOXD-AS1 and the clinicopathological characteristics of patients with HCC, no significances were observed between HOXD-AS1 and the tumor size, age and gender of patients (Additional file 1: Table S1). All these findings suggested that HOXD-AS1 was up-regulated in HCC tissue and was related with the progression of HCC.

### HOXD-AS1 increases HCC cell growth

**Fig. 2:**
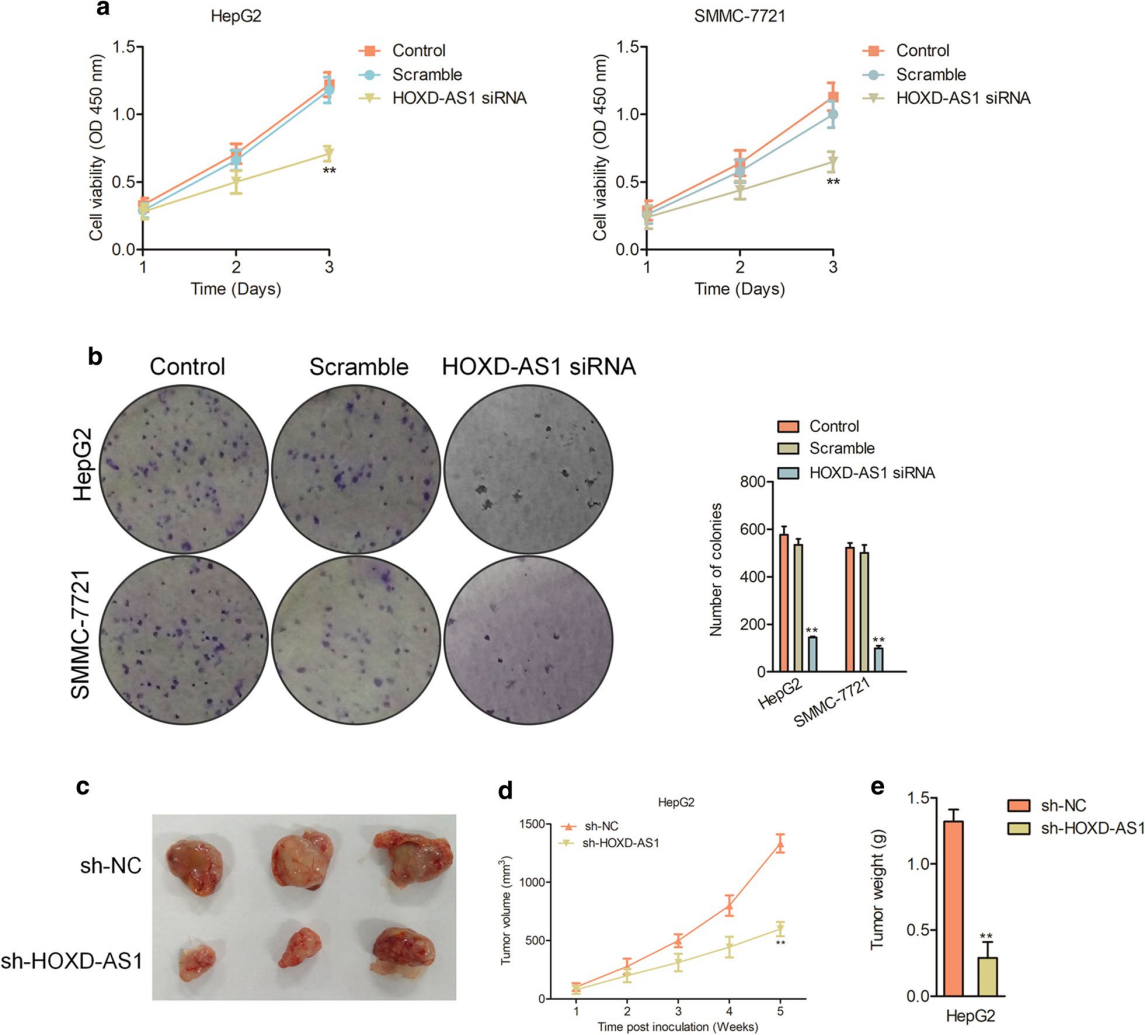
Knockdown of HOXD-AS1 suppresses HCC cell growth. ** a** CCK-8 assay was used to analysis the viability in HOXD-AS1 siRNA or scramble transfected HCC cell. ** b** Colony formation assay using HOXD-AS1 siRNA or scramble transfected HCC cell. ^**^P<0.01 compared to control. ** c** The stable HOXD-AS1 knockdown HepG2 cells were inoculated into nude mice. The tumor tissues form the two groups were shown. ** d** The tumor volume was measured each week and the tumor growth curve was calculated. ** e** The tumor weight in the two groups. ^**^P<0.01 compared to sh-NC

To elaborate the influence of HOXD-AS1 on HCC cell growth, HOXD-AS1 siRNA was transfected into SMMC-7721 or HepG2 cells, which presented with relatively higher level of HOXD-AS1 to decrease the level of HOXD-AS1 (Additional file 1: Figure S2a). Meanwhile, pcDNA3.1 vector containing HOXD-AS1 was transfected into BEL7402 or HuH7 cells to increase the level of HOXD-AS1 and qPCR was conducted to confirm the transfection efficiencies (Additional file 1: Figure 3a). Next, we observed that downregulation of HOXD-AS1 decreased the cell viabilities of HepG2 and SMMC-7721 cells (Fig. 2a) whereas overexpression of HOXD-AS1 promoted the proliferation of BEL7402 and HuH7 cells (Additional file 1: Figure S3b). In addition, the colony formation test shown that downregulation of HOXD-AS1 significantly reduced the colony formation abilities of HepG2 and SMMC-7721 cells (Fig. 2b), while HOXD-AS1 upregulation induced the colony formation of BEL7402 or HuH7 cells (Additional file 1: Figure 3c). Consistently, downregulation of HOXD-AS1 significantly inhibited the proliferation and colony formation abilities of BEL7402 or HuH7 cells (Additional file 1: Figure S4a–c). To elaborate the effect of HOXD-AS1 on HCC cell growth in nude mice, stable HOXD-AS1 knockdown HepG2 cell line was constructed (sh-HOXD-AS1). Then, HepG2 cells were injected subcutaneously into nude mice. As shown in Fig. 2c-e, both the tumor volume and weight were markedly decreased in sh-HOXD-AS1 group in contrast to sh-NC group. Finally, the qPCR assay implied that HOXD-AS1 levels were suppressed in tumors that were formed by sh-HOXD-AS1 transfected HepG2 cells (Additional file 1: Figure S2b).

**Fig. 3:**
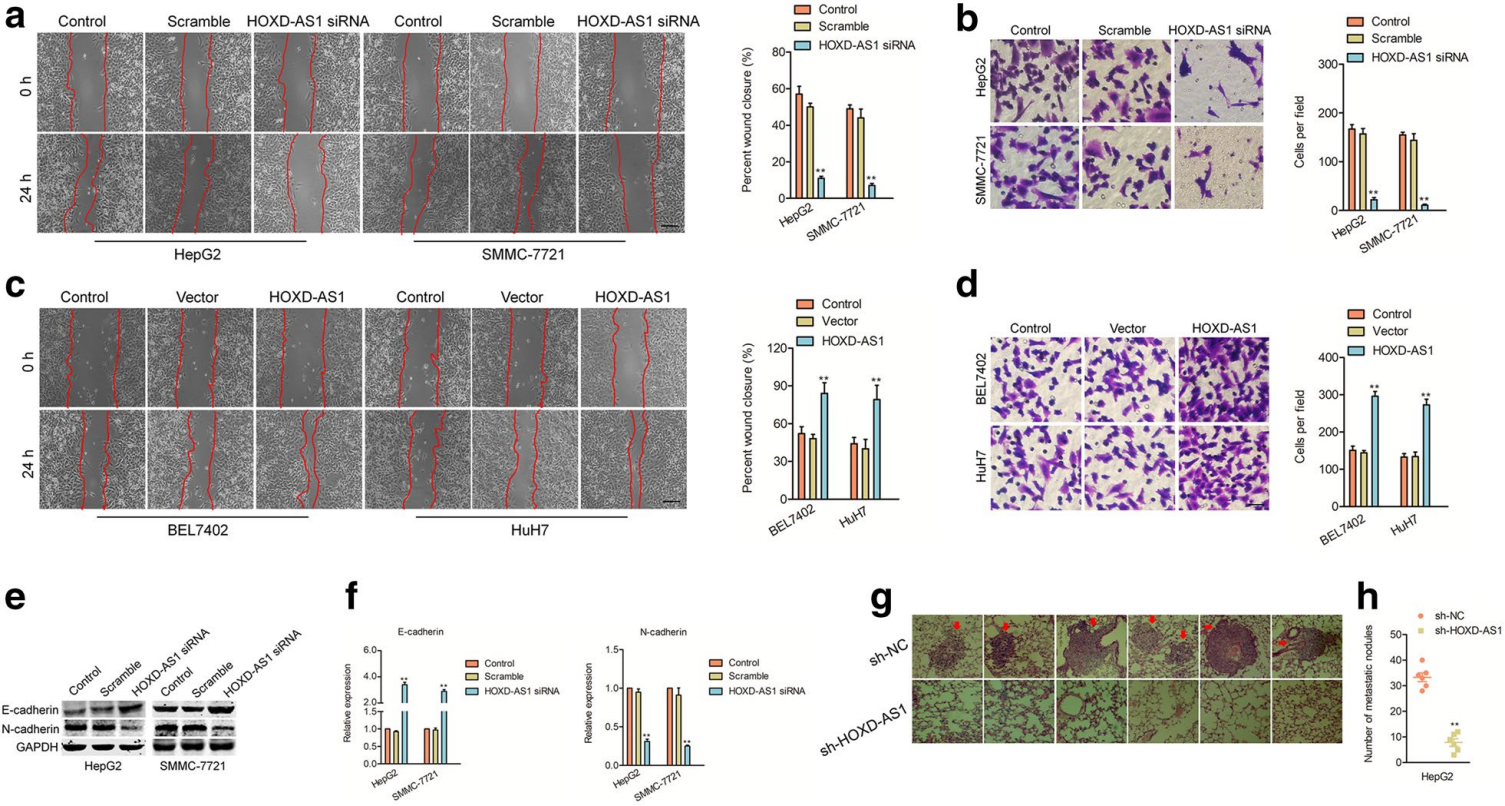
Knockdown of HOXD-AS1 inhibits the metastasis of HCC cell. A. The migration of HepG2 and SMMC-7721 cells after transfection of HOXD-AS1 siRNA was detected using wound healing assay. B. The invasion abilities of HepG2 and SMMC-7721 cells after transfection of HOXD-AS1 siRNA were detected by Transwell assay. C. The migration of BEL7402 and HuH7 cells after ectopic expression of HOXD-AS1 was measured using wound healing assay. D. The invasion abilities of BEL7402 and HuH7 cells after ectopic expression of HOXD-AS1 were analyzed. E. Downregulation of HOXD-AS1 increased the level of E-cadherin while reduced the level of N-cadherin. F. Downregulation of HOXD-AS1 increased the mRNA level of E-cadherin while reduced the mRNA level of N-cadherin. ^**^P<0.01 compared to control. G. The stable HOXD-AS1 knockdown HepG2 cells were injected into nude mice, the micro-metastasis in the lung from the two groups were shown. H. The numbers of lung metastasis were quantified and showed by each data point. ^**^P<0.01 compared to sh-NC

**Fig. 4:**
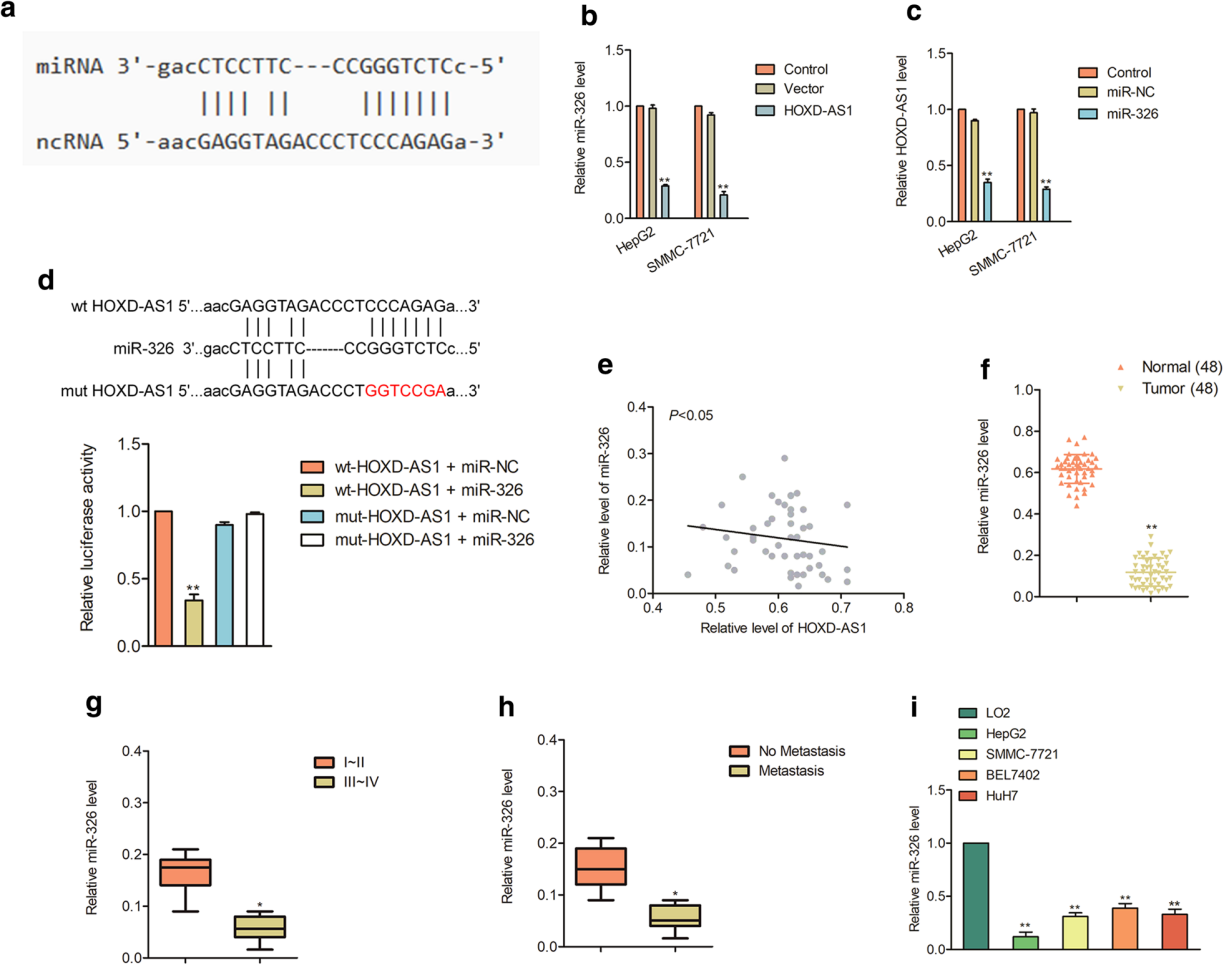
HOXD-AS1 binds to miR-326. ** a** The potential binding sites were identified between HOXD-AS1 and miR-326 using bioinformatics analysis tool, starbase v2.0 (http://starbase.sysu.edu.cn/mirLncRNA.php). ** b** Over-expression of HOXD-AS1 inhibited the levels of miR-326 in SMMC-7721 and HepG2 cells. ** c** Cells were transfected with miR-NC or miR-326, and miR-326 transfection remarkably suppressed the levels of HOXD-AS1 in SMMC-7721 and HepG2 cells. ** d** Luciferase reporter analysis suggested that HOXD-AS1 directly targeted miR-326. ** e** The relationship between HOXD-AS1 and miR-326 in HCC tissues was evaluated. ** f** The level of miR-326 was measured by qPCR method in 48 cases of HCC and adjacent non-cancerous tissues. ^**^P<0.01 compared to normal. ** g** Representative association between miR-326 and I-II or III-IV stage was shown. ^*^P<0.05 compared to I-II. ** h** Relative levels of miR-326 in patients with distant metastasis or without metastasis. ^*^P<0.05 compared to no metastasis. ** i** The level of miR-326 in four HCC cells and LO2 as detected by qPCR analysis. ^**^P<0.01 compared to LO2 cell

### HOXD-AS1 promotes HCC cell aggressiveness and metastasis

For exploring the impact of HOXD-AS1 on the migration and invasion of HCC cell, the wound healing and transwell invasion assays were conducted. The migration assay indicated that downregulation of HOXD-AS1 remarkably restrained the migration abilities of SMMC-7721 and HepG2 cells (Fig. 3a). Furthermore, the transwell invasion test indicated that downregulation of HOXD-AS1 weakened the invasion abilities in HepG2 and SMMC-7721 cells (Fig. 3b). Consistently, downregulation of HOXD-AS1 also significantly inhibited the migration and invasion abilities of BEL7402 or HuH7 cells (Additional file 1: Figure S4d–e). On the contrary, overexpression of HOXD-AS1 enhanced the migration and invasiveness of BEL7402 and HuH7 cells (Fig. 3c–d). The epithelial to mesenchymal transition (EMT) is a multistep, plastic and reversible process that allows epithelial cells to acquire mesenchymal characteristics. An increasing number of studies support the role of lncRNAs in the regulation of tumor progression and metastasization through the regulation of EMT [5, 15, 16]. Herein, we observed that downregulation of HOXD-AS1 elevated the level of E-cadherin (epithelial marker) and decreased the expression of mesenchymal marker, N-cadherin (Fig. 3e–f). To future analysis the effect of HOXD-AS1 on the metastatic ability of HCC cell in vivo, sh-NC or sh-HOXD-AS1 transfected HepG2 cells were injected into nude mice. As shown in Fig. 3g–h, the micrometastatic nodules in lung tissues were markedly decreased in nude mice that were injected with sh-HOXD-AS1 transfected HepG2 cells when compared with mice that were injected with sh-NC transfected cells. All findings implied that HOXD-AS1 facilitated the mobility, invasiveness, and metastasis of HCC cell.

### HOXD-AS1 binds with miR-326

To uncover the underlying mechanism by which HOXD-AS1 in regulating the development of HCC, bioinformatics analysis tool, starbase v2.0 (http://starbase.sysu.edu.cn/mirLncRNA.php) was chose to find the putative targets of HOXD-AS1 [17]. The miRNAs (total twenty) that formed complementary base pairing with HOXD-AS1 were shown in Additional file 1: Figure S5a. We then examined the expression of these miRNAs in response to HOXD-AS1 overexpression. As found in Additional file 1: Figure S5b, there was a list of miRNAs that were downregulated in response to HOXD-AS1. We focused on miR-326, which was of the greatest fold-change in response to HOXD-AS1 in both HepG2 and SMMC-7721 cells. As shown in Fig. 4a, there were ten binding sites between miR-326 and HOXD-AS1. Then, we analyzed the expressions of miR-326 in HOXD-AS1 transfected HCC cell. As showed in Fig. 4b, the levels of miR-326 were decreased in HepG2 and SMMC-7721 cells after HOXD-AS1 transfection. Next, HepG2 or SMMC-7721 cells were transfected miR-326 mimics and we noticed that transfection of miR-326 reduced the levels of HOXD-AS1 (Fig. 4c). The luciferase reporter gene analysis was carried out to confirm that HOXD-AS1 directly bind to miR-326. The wild type (wt) sequence of HOXD-AS1 containing the binding sites of miR-326 (wt-HOXD-AS1) or mutant type (mut) fragment (mut-HOXD-AS1) of HOXD-AS1 was inserted into luciferase reporter plasmid. HEK-293T cells were cotransfected with the plasmid combination with miR-NC or miR-326. Luciferase activity was degraded by miR-326 in wt-HOXD-AS1 transfected HEK-293T cells. Nevertheless, the luciferase activity in mut-HOXD-AS1 transfected HEK-293T cells was not effectively inhibited by miR-326 (Fig. 4d). Importantly, we found that miR-326 level was inversely related with the level of HOXD-AS1 in clinical HCC tissues (Fig. 4e). To analysis the expression of miR-326 in HCC, we determined the levels of miR-326 in 48 cases of HCC and non-cancer tissues by using qPCR analysis. As showed in Fig. 4f, miR-326 was remarkably downexpressed in HCC than that in normal tissues. Moreover, the higher level of miR-326 was negatively associated with the metastasis (Fig. 4g) and advanced stages of HCC (Fig. 4H). We then investigated the relationship between the level of miR-326 and the clinicopathological characteristics of patients with HCC, no association was observed between miR-326 and age, gender, and tumor size (Additional file 1: Table S2). Finally, the expressions of HOXD-AS1 among LO2 and HCC cells were detected by using qPCR test. As showed in Fig. 4i, miR-326 was downexpressed in HCC cells than that in LO2 cell.

**Fig. 5:**
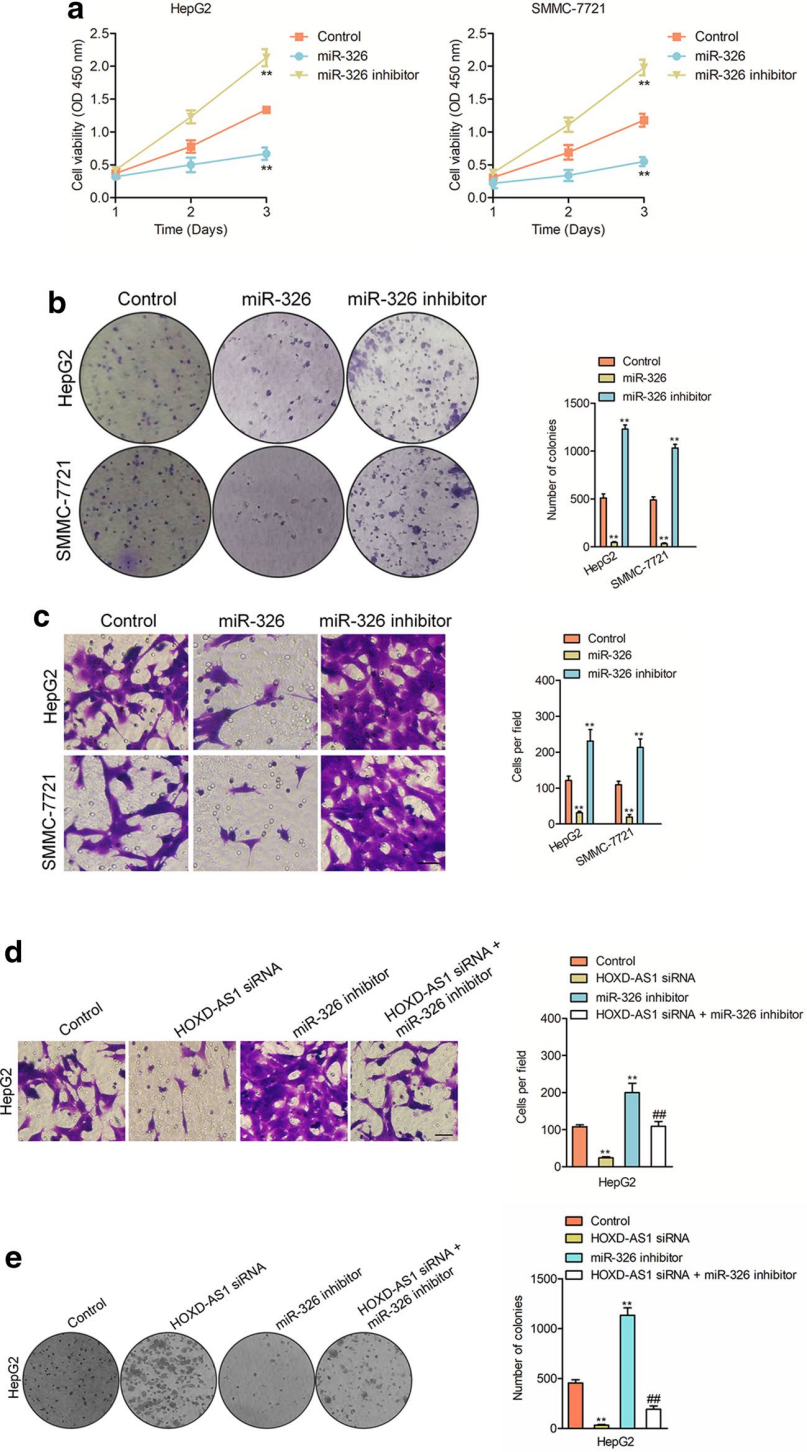
The effects of miR-326 on HCC cell. ** a** CCK-8 assay showed that the proliferation of HepG2 and SMMC-7721 was inhibited by miR-326 mimics and stimulated by miR-326 inhibitor. ** b** The colony formation abilities were suppressed in HepG2 and SMMC-7721 cells by miR-326 mimics and stimulated by miR-326 inhibitor. ** c** The invasion capacities were inhibited in HepG2 and SMMC-7721 cells by miR-326 and stimulated by miR-326 inhibitor. ^**^P< 0.01 compared to control. ** d** Transwell assays in HepG2 cells after transfected with HOXD-AS1 siRNA, miR-326 inhibitor or both. ** e** Colony formation assays in HepG2 cells after transfected with HOXD-AS1 siRNA, miR-326 inhibitor or both. ^**^P< 0.01 compared to control, ^##^*P*< 0.01 compared to miR-326 inhibitor

### The biological activities of HOXD-AS1 are modulated by miR-326

To future investigate the potential role of miR-326 in HCC, SMMC-7721 or HepG2 cells were transfected with miR-326 or miR-326 inhibitor (Additional file 1: Figure S6a). The proliferation test suggested that miR-326 overexpression restrained the proliferation while silencing of miR-326 enhanced HepG2 and SMMC-7721 cells proliferation (Fig. 5a). Furthermore, miR-326 transfection inhibited the colony formation as well as invasive abilities of and SMMC-7721 and HepG2 cells while silencing of miR-326 caused opposites results (Fig. 5b–c). In order to prove whether miR-326 was involved in HOXD-AS1 mediated the biological activities in HCC cell, HepG2 cells were cotransfected with miR-326 inhibitor and HOXD-AS1 siRNA (Additional file 1: Figure S6b). We observed that the inhibitory effects of HOXD-AS1 knockdown on the invasion and colony formation of HepG2 cells were rescued by the miR-326 inhibitor cotransfection (Fig. 5d–e). However, overexpressing miR-326 in HOXD-AS1 overexpressed cell reversed the promoted effect of HOXD-AS1 on invasion and colony formation (Additional file 1: Figure S7). All findings indicated that HOXD-AS1 regulated the aggressive phenotypes of HCC cell at least partly through modulating miR-326.

**Fig. 6:**
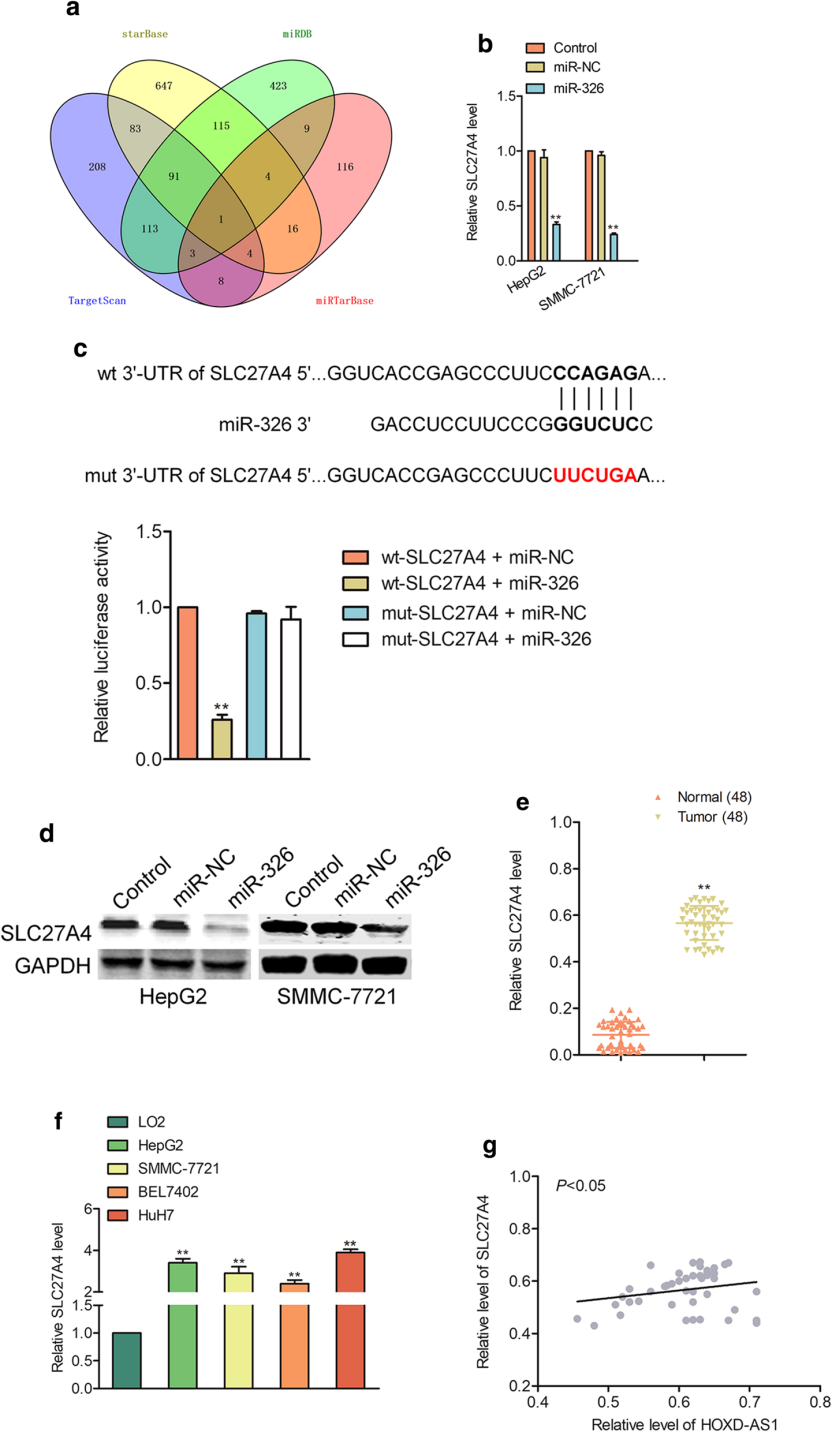
SLC27A4 is regulated by HOXD-AS1 and miR-326. ** a** Venn graph represented the candidate common target genes determined by four bioinformatics analysis tool (TargetScan, miRTarBase, StarBase and miRDB). ** b** HepG2 or SMMC-7721 cells were transfected with miR-NC or miR-326 and the levels of SLC27A4 was measured by qPCR assay. ^**^P< 0.01 compared to control. ** c** The complementary sequences of miR-326 were discovered in 3’-UTR of SLC27A4 mRNA using TargetScan. The mutagenesis was performed in the complementary sites for the seed region of miR-326 (wt, wild type; mut, mutant type). MiR-326 inversely modulated the luciferase activity of plasmids that containing wt 3’-UTR of SLC27A4. ^**^P< 0.01 compared to miR-NC + wt-SLC27A4. ** d** HepG2 and SMMC-7721 cells were transfected with miR-326 or miR-NC. Western blotting assay indicated that up-regulation of miR-326 decreased the expression of SLC27A4. ** e** The levels of SLC27A4 in HCC tissues and adjacent normal tissues were measured by qPCR. ^**^P< 0.01 compared to normal. ** f** The levels of SLC27A4 in four HCC cells and LO2 were detected by qPCR analysis. ^**^P< 0.01 compared to LO2 cell. ** g** The association between SLC27A4 and HOXD-AS1 in HCC tissues was evaluated

**Fig. 7:**
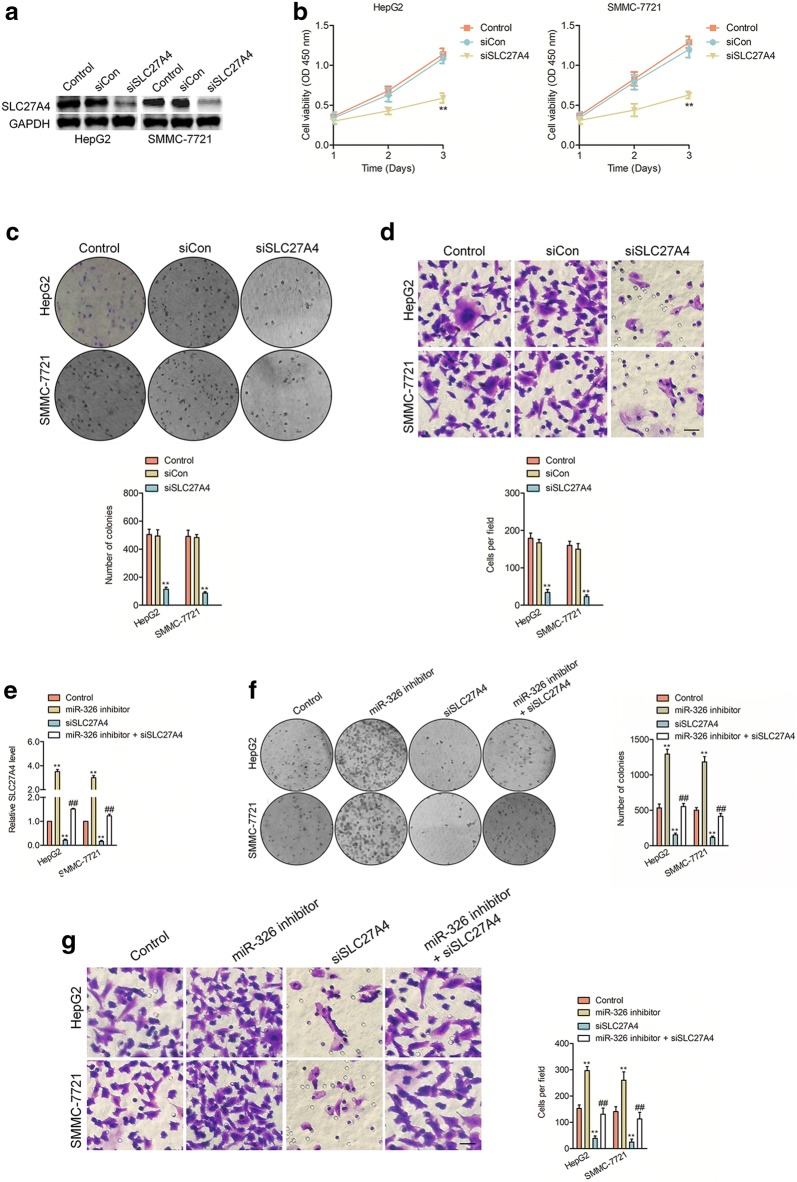
The effects of SLC27A4 on HCC cell. ** a** The levels of SLC27A4 in HepG2 and SMMC-7721 cells were reduced by knockdown of SLC27A4. ** b** The proliferation of siSLC27A4 or control siRNA transfected HepG2 and SMMC-7721 cells were measured by CCK-8 assay. ** c** The colony formation of siSLC27A4 or control siRNA transfected HepG2 and SMMC-7721 cells. ** d** The invasion abilities of HepG2 and SMMC-7721 cells that were transfected with siSLC27A4 or control siRNA. ^**^P< 0.01 compared to control.** e** The level of SLC27A4 in HepG2 and SMMC-7721 cells were measured by qPCR assay after transfected with siSLC27A4, miR-326 inhibitor or both. ** f** The growth of HepG2 and SMMC-7721 cells were measured by colony formation assay after transfected with siSLC27A4, miR-326 inhibitor or both. ** g** The invasion abilities of HepG2 and SMMC-7721 cells after transfected with siSLC27A4, miR-326 inhibitor or both. ^**^P< 0.01 compared to control, ^##^*P*< 0.01 compared to siSLC27A4

### SLC27A4 is the target of miR-326

Next, we used miRDB, starBase, TargetScan and miRTarBase to predict the potential target genes of miR-326 [17–20]. The common target gene (SLC27A4) which was obtained from the four bioinformatics analysis tools were summarized in Fig. 6a. Next, qPCR was used to analyze the expressions of SLC27A4 in miR-326 transfected HepG2 or SMMC-7721 cells. As showed in Fig. 6b, the level of SLC27A4 was markedly decreased by miR-326 in both HepG2 and SMMC-7721 cells. To confirm that miR-326 directly bound to SLC27A4, the luciferase reporter gene analysis was performed. As showed in Fig. 6c, the luciferase activity in wt-SLC27A4 and miR-326 mimics cotransfected HEK-293T cell was reduced whereas the luciferase activity in HEK-293T cell which was transfected with mut-SLC27A4 was not obviously reduced by miR-326. Finally, we detected the role of miR-326 on the expression of SLC27A4 using western blotting assay. As showed in Fig. 6d, miR-326 over-expression reduced the expression of SLC27A4 in HepG2 or SMMC-7721 cells. Furthermore, we observed that SLC27A4 was upregulated in HCC tissues when compared to adjacent normal tissues (Fig. 6e). Consistently, SLC27A4 was obviously upregulated in HCC cell lines (HepG2, SMMC-7721, BEL7402 and HuH7) than that in normal hepatocytes cell line, LO2 (Fig. 6f). Additional, we demonstrated a positive relationship between HOXD-AS1 and SLC27A4 in HCC tissues (Fig. 6g). As expected, a negative association was found between miR-326 and SLC27A4 in HCC tissues (Additional file 1: Figure S8). All findings indicated that HOXD-AS1 regulated the expression of SLC27A4.

### SLC27A4 is overexpressed in HCC

To future investigate the function of SLC27A4 in HCC cells, SMMC-7721 and HepG2 cells were transfected siSLC27A4 to decrease the level of SLC27A4. Western blotting analysis confirmed the knockdown efficiency of SLC27A4 in two cell lines (Fig. 7a). Then, we observed that downregulation of SLC27A4 markedly inhibited the proliferation of HepG2 and SMMC-7721 cells (Fig. 7b). Likewise, siSLC27A4 decreased the invasiveness and clonogenic abilities of SMMC-7721 and HepG2 cells (Fig. 7c–d). Finally, we observed that the colony formation and invasiveness which were promoted by miR-326 inhibitor could be reversed by downregulation of SLC27A4 in HepG2 and SMMC-7721 cells (Fig. 7e–g).

## Discussion

Increasing evidences have verified that lncRNAs exert critical functions in the tumorigenesis and progress of malignant tumors. Previous investigations have verified that HOXD-AS1 promotes the development of multiple tumor types, including colorectal cancer, melanoma and ovarian cancer [21–23]. In our study, we proved that lncRNA HOXD-AS1 was strikingly overexpressed in human HCC and was allied to the aggressive tumor phenotypes (metastasis and advanced stage) of patients with HCC. The functional assays proved that HOXD-AS1 promoted the growth, migration and invasion as well as metastasis of HCC cell in vitro and in vivo. All these findings implied that HOXD-AS1 played a crucial oncogenic role in HCC and could be a potential predictor for the prognosis of patient with HCC.

Recent reports suggest that lncRNAs are frequently involved into the competing endogenous RNAs (ceRNAs) network, where lncRNAs modulate the expression of miRNA target genes through binding with miRNAs [24]. For instance, lncRNA TCONS_00026907 participates into the development of cervical carcinoma by reducing the level of miR-143-5p [25]. LncRNA 00511 acts as an oncogene in non-small-cell lung cancer (NSCLC) via binding to EZH2 and suppressing p57 [26]. In this research, we observed that HOXD-AS1 was high-expressed in human HCC tissues and HCC cells. The online bioinformatics analysis combination with luciferase activity assays demonstrated that HOXD-AS1 bound with miR-326. Previous study proves that miRNA-326 restrains the growth and aggressiveness of cervical carcinoma cell by mediating ETS Transcription Factor ELK1 (ELK1) [27]. Moreover, miR-326 weakens cell proliferation via lowering NOB1 level in gastric cancer [28]. Consistently, our study revealed that miR-326 was downexpressed in human HCC and proved that miR-326 repressed the malignant phenotypes of HCC cell.

In general, lncRNAs exert their functions via serving as ceRNAs and modulating the expression of miRNA target gene [29]. Herein, the analysis using bioinformatics prediction tools suggested that SLC27A4 was the target of miR-326 and the luciferase reporter gene test certified that miR-326 binding the 3′-UTR of SLC27A4. SLC27A4 has been proved to be dysregulated in cancers and is involved into the regulation of cancer progression. In breast cancer, SLC27A4 enhances the cell growth, migration, and invasion of Hs578T and MDA-MB-231 [30]. SLC27A4 is also overexpressed in lung cancer cell lines as well as lung tumor tissues [31]. Consistent with previous reports, we observed that SLC27A4 was markedly overexpressed in HCC tissues and cells. Meanwhile, downregulation of SLC27A4 suppressed the growth, migrate and invasive capacities of HCC cell. EMT causes cancer cell to lose their epithelial characteristics, acquire migratory and invasive ability and away from epithelial cell community to become mesenchymal cells [32, 33]. A growing body of research suggests that the role of lncRNAs in the regulation of tumor progression and metastasization through the regulation of EMT. In the current study, we proved that HOXD-AS1 silencing increased the protein expressions and mRNA levels of epithelial marker, E-cadherin and suppressed the expression of mesenchymal marker, N-cadherin in HCC cell. All these findings indicated that downregulation of HOXD-AS1 restrained the EMT process of human HCC cell. In addition, the cell growth and invasion abilities enhanced by miR-326 inhibition were partly neutralized by downregulation of SLC27A4, which suggest that SLC27A4 was vital for the miR-326 regulating the biological phenotypes of HCC cell.

## Conclusion

In conclusion, we identify that HOXD-AS1 facilitates the growth, invasiveness and metastatic phenotypes of HCC cell by binding with miR-326 thereby modulating the level of SLC27A4. The current research illuminates the precise function of HOXD-AS1/miRNA-326/SLC27A4 network in HCC, and indicates that HOXD-AS1 might be developed as a potential target for HCC.

## Supplementary information

**Additional file 1: Figure S1.** The overall survival of HCC patients between the low and high HOXD-AS1 expression group was compared.** Figure S2.**A. HepG2 and SMMC-7721 cell was transfected with HOXD-AS1 siRNA and the level of HOXD-AS1 was determined by qPCR. **P<0.01 compared to control. B. The level of HOXD-AS1 in tumor tissues formed by sh-HOXD-AS1 or sh-NC transfected cells was analyzed using qPCR. **P<0.01 compared to sh-NC. ** Figure S3.** The effects of HOXD-AS1 on HCC cell growth and colony formation. A. The level of HOXD-AS1 was determined by qPCR in BEL7402 and HuH7 cells after transfection with HOXD-AS1 or control vector. B. CCK-8 assay showed that the proliferation of BEL7402 and HuH7 was stimulated by HOXD-AS1. C. The colony formation abilities in BEL7402 and HuH7 cells were stimulated by HOXD-AS1. *P<0.01 compared to control.** Figure S4.** The effects of HOXD-AS1 on HCC cell growth, migration and invasion. A. The level of HOXD-AS1 was determined by qPCR in BEL7402 and HuH7 cells after transfection with HOXD-AS1 siRNA. B. CCK-8 assay was used to analysis the viability in HOXD-AS1 siRNA or scramble transfected HCC cell. C. Colony formation assay using HOXD-AS1 siRNA or scramble transfected HCC cell. D. The migration of BEL7402 and HuH7 cells after transfection of HOXD-AS1 siRNA was detected using wound healing assay. E. The invasion abilities of HepG2 and SMMC-7721 cells after transfection of HOXD-AS1 siRNA were detected by transwell assay. *P<0.01 compared to control.** Figure S5.** A. The potential targets of HOXD-AS1 were identified using bioinformatics analysis tool, starbase v2.0 (http://starbase.sysu.edu.cn/mirLncRNA.php). B. The miRNAs that were downregulated in response to HOXD-AS1 overexpression in both HepG2 and SMMC-7721 cells.** Figure S6.** A. The levels of miR-326 were determined by qPCR in HepG2 and SMMC-7721 cells after transfection with miR-326 mimics, miR-326 inhibitor or control miRNA. B. HepG2 cells were transfected with HOXD-AS1 siRNA, miR-326 inhibitor or both and the level of miR-326 was detected using qPCR assay. **P<0.01 compared to control, ##P<0.01 compared to miR-326 inhibitor. ** Figure S7.**The promoted effect of HOXD-AS1 on colony formation and invasion could be reversed by miR-326 in HepG2 and SMMC-7721 cells. A. The level of HOXD-AS1 in HepG2 cells was measured by qPCR assay after transfected with HOXD-AS1, miR-326 or both. B. The growth of HepG2 cell was measured by colony formation assays after transfected with HOXD-AS1, miR-326 or both. C. The invasion abilities of HepG2 cell after transfected with HOXD-AS1, miR-326 or both. **P<0.01 compared to control, ##P<0.01 compared to miR-326.** Figure S8.** The association between SLC27A4 and miR-326 in HCC tissues was evaluated by qPCR assay. **Table S1.** Association of lncRNA HOXD-AS1 expression with clinicopathologic features in patients with HCC. Table 2. Association of miR-326 expression with clinicopathologic features in patients with HCC.

## Data Availability

The datasets used in this study are available from the corresponding author upon reasonable request.

## Abbreviations

lncRNA: long non-coding RNA
HOXD-AS1: HOXD cluster antisense RNA 1
qPCR: quantitative real-time PCR
HCC: hepatocellular carcinoma
SLC27A4: solute carrier family 27 member 4
ceRNAs: competing endogenous RNAs
