# Supplementary material for: LncRNA HOXD-AS1 promotes the metastasis of human hepatocellular carcinoma via modulating miR-326/SLC27A4
Source: Cancer Cell Int. 2020 May 12;20:161. doi: 10.1186/s12935-020-01217-8 (PMC7216491; doi:10.1186/s12935-020-01217-8)
Supplement: Supplementary file 1 — Additional file 1: Figure S1. The overall survival of HCC patients between the low and high HOXD-AS1 expression group was compared. Figure S2.A. HepG2 and SMMC-7721 cell was transfected with HOXD-AS1 siRNA and the level of HOXD-AS1 was determined by qPCR. **P<0.01 compared to control. B. The level of HOXD-AS1 in tumor tissues formed by sh-HOXD-AS1 or sh-NC transfected cells was analyzed using qPCR. **P<0.01 compared to sh-NC. Figure S3. The effects of HOXD-AS1 on HCC cell growth and colony formation. A. The level of HOXD-AS1 was determined by qPCR in BEL7402 and HuH7 cells after transfection with HOXD-AS1 or control vector. B. CCK-8 assay showed that the proliferation of BEL7402 and HuH7 was stimulated by HOXD-AS1. C. The colony formation abilities in BEL7402 and HuH7 cells were stimulated by HOXD-AS1. *P<0.01 compared to control. Figure S4. The effects of HOXD-AS1 on HCC cell growth, migration and invasion. A. The level of HOXD-AS1 was determined by qPCR in BEL7402 and HuH7 cells after transfection with HOXD-AS1 siRNA. B. CCK-8 assay was used to analysis the viability in HOXD-AS1 siRNA or scramble transfected HCC cell. C. Colony formation assay using HOXD-AS1 siRNA or scramble transfected HCC cell. D. The migration of BEL7402 and HuH7 cells after transfection of HOXD-AS1 siRNA was detected using wound healing assay. E. The invasion abilities of HepG2 and SMMC-7721 cells after transfection of HOXD-AS1 siRNA were detected by transwell assay. *P<0.01 compared to control. Figure S5. A. The potential targets of HOXD-AS1 were identified using bioinformatics analysis tool, starbase v2.0 (http://starbase.sysu.edu.cn/mirLncRNA.php). B. The miRNAs that were downregulated in response to HOXD-AS1 overexpression in both HepG2 and SMMC-7721 cells. Figure S6. A. The levels of miR-326 were determined by qPCR in HepG2 and SMMC-7721 cells after transfection with miR-326 mimics, miR-326 inhibitor or control miRNA. B. HepG2 cells were transfected with HOXD-AS1 siRNA, miR-326 inhibitor [file 12935_2020_1217_MOESM1_ESM.docx]

**
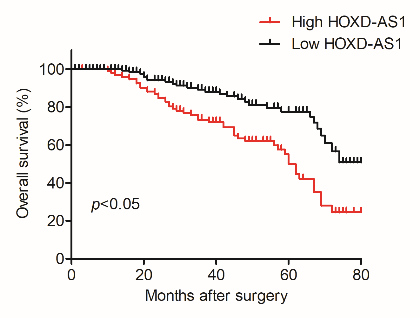
**

**Supplementary Figure S1.** The overall survival of HCC patients between the low and high HOXD-AS1 expression group was compared.

**
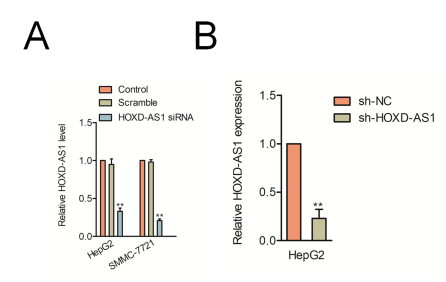
**

**Supplementary Figure S2. A.** HepG2 and SMMC-7721 cell was transfected with HOXD-AS1 siRNA and the level of HOXD-AS1 was determined by qPCR. ^**^*P*<0.01 compared to control. **B.** The level of HOXD-AS1 in tumor tissues formed by sh-HOXD-AS1 or sh-NC transfected cells was analyzed using qPCR. ^**^*P*<0.01 compared to sh-NC.

**
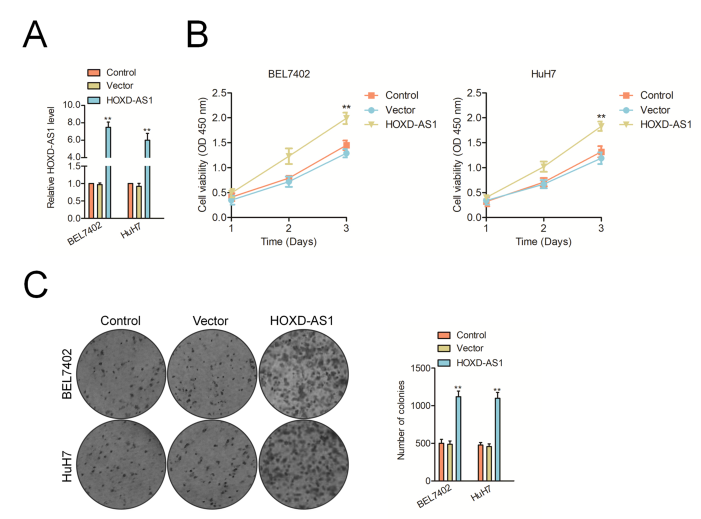
**

**Supplementary Figure S3. The effects of HOXD-AS1 on HCC cell growth and colony formation.** **A.** The level of HOXD-AS1 was determined by qPCR in BEL7402 and HuH7 cells after transfection with HOXD-AS1 or control vector. **B.** CCK-8 assay showed that the proliferation of BEL7402 and HuH7 was stimulated by HOXD-AS1. **C.** The colony formation abilities in BEL7402 and HuH7 cells were stimulated by HOXD-AS1. ^*^*P*<0.01 compared to control.

**
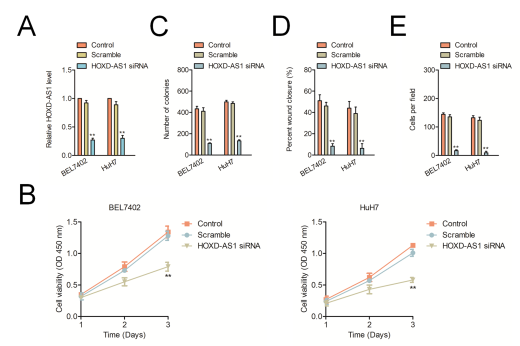
**

**Supplementary Figure S4. The effects of HOXD-AS1 on HCC cell growth, migration and invasion.** **A.** The level of HOXD-AS1 was determined by qPCR in BEL7402 and HuH7 cells after transfection with HOXD-AS1 siRNA. **B.** CCK-8 assay was used to analysis the viability in HOXD-AS1 siRNA or scramble transfected HCC cell. **C.** Colony formation assay using HOXD-AS1 siRNA or scramble transfected HCC cell. **D.** The migration of BEL7402 and HuH7 cells after transfection of HOXD-AS1 siRNA was detected using wound healing assay. **E.** The invasion abilities of HepG2 and SMMC-7721 cells after transfection of HOXD-AS1 siRNA were detected by transwell assay. ^*^*P*<0.01 compared to control.

**
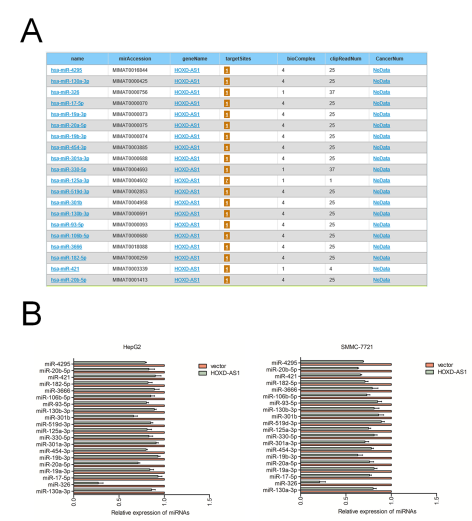
**

**Supplementary Figure S5. A.** The potential targets of HOXD-AS1 were identified using bioinformatics analysis tool, starbase v2.0 (<http://starbase.sysu.edu.cn/mirLncRNA.php>). **B.** The miRNAs that were downregulated in response to HOXD-AS1 overexpression in both HepG2 and SMMC-7721 cells.

**
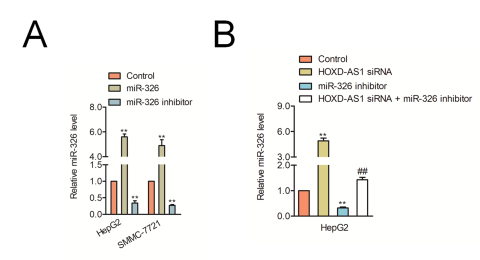
**

**Supplementary Figure S6. A.** The levels of miR-326 were determined by qPCR in HepG2 and SMMC-7721 cells after transfection with miR-326 mimics, miR-326 inhibitor or control miRNA. **B.** HepG2 cells were transfected with HOXD-AS1 siRNA, miR-326 inhibitor or both and the level of miR-326 was detected using qPCR assay. ^**^*P*<0.01 compared to control, ^##^*P*<0.01 compared to miR-326 inhibitor.


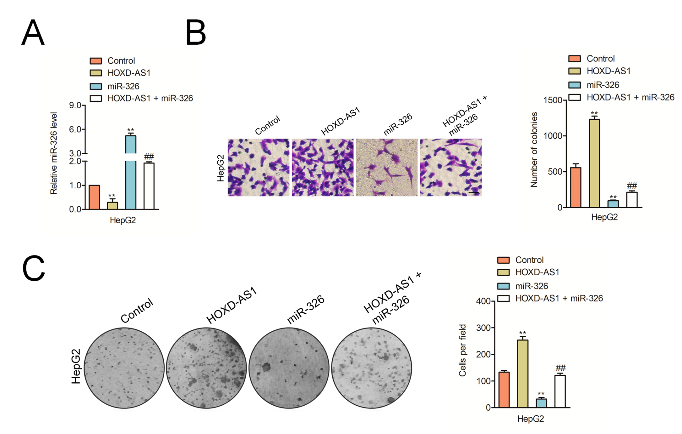


**Supplementary Figure S7. The promoted effect of HOXD-AS1 on colony formation and invasion could be reversed by miR-326 in HepG2 and SMMC-7721 cells. A.** The level of HOXD-AS1 in HepG2 cells was measured by qPCR assay after transfected with HOXD-AS1, miR-326 or both. **B.** The growth of HepG2 cell was measured by colony formation assays after transfected with HOXD-AS1, miR-326 or both. **C.** The invasion abilities of HepG2 cell after transfected with HOXD-AS1, miR-326 or both. ^**^*P*<0.01 compared to control, ^##^*P*<0.01 compared to miR-326.

**
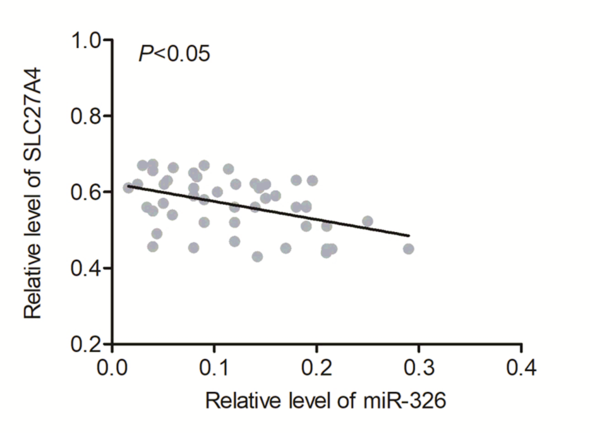
**

**Supplementary Figure S8.** The association between SLC27A4 and miR-326 in HCC tissues was evaluated by qPCR assay.

**Supplementary Table S1**. Association of lncRNA HOXD-AS1 expression with clinicopathologic features in patients with HCC.

| **Clinical parameter** | **HOXD-AS1** | | ***P*-value** |
| --- | --- | --- | --- |
|  | **High** | **Low** |  |
| **Age (years)** |  |  | 0.331 |
| ≤60 | 17 | 10 |  |
| >60 | 15 | 6 |  |
| **Gender** |  |  | 0.563 |
| Female | 14 | 8 |  |
| Male | 18 | 8 |  |
| **Tumor size (cm)** |  |  | 0.164 |
| ≥5 | 16 | 6 |  |
| <5 | 16 | 10 |  |
| **Clinical stage (TNM)** |  |  | 0.023 |
| I~II | 11 | 12 |  |
| III~IV | 21 | 4 |  |
| **Distant metastasis** |  |  | 0.021 |
| Yes | 26 | 2 |  |
| No | 6 | 14 |  |

**Supplementary Table S2**. Association of miR-326 expression with clinicopathologic features in patients with HCC.

| **Clinical parameter** | **miR-326** | | ***P*-value** |
| --- | --- | --- | --- |
|  | **High** | **Low** |  |
| **Age (years)** |  |  | 0.271 |
| ≤60 | 9 | 18 |  |
| >60 | 8 | 13 |  |
| **Gender** |  |  | 0.112 |
| Female | 10 | 12 |  |
| Male | 7 | 19 |  |
| **Tumor size (cm)** |  |  | 0.204 |
| ≥5 | 10 | 12 |  |
| <5 | 7 | 19 |  |
| **Clinical stage (TNM)** |  |  | 0.019 |
| I~II | 12 | 11 |  |
| III~IV | 5 | 20 |  |
| **Distant metastasis** |  |  | 0.036 |
| Yes | 3 | 25 |  |
| No | 14 | 6 |  |
